# Supplementary material for: Large Scale Subject Category Classification of Scholarly Papers With Deep Attentive Neural Networks
Source: Front Res Metr Anal. 2021 Feb 10;5:600382. doi: 10.3389/frma.2020.600382 (PMC8025978; doi:10.3389/frma.2020.600382)
Supplement: Supplementary file 1 [file datasheet1.pdf]

Supplementary Material

1 SUPPLEMENTARY DATA

Table S1. Comparison of models used in the first-level classifier which classifies abstracts into 81 SCs. For column $\mathbf{D}_{\mathrm{WE}}$ the integer in the parentheses is the dimension of the retrained word vectors. **#Nr** is the number of neurons per layer. **#Lr** is the number of hidden layers. mi$\mathbf{F_1}$ stands for micro-F_1. $\mathbf{T}_{\mathrm{t}}$ is the time elapsed in seconds to classify 638k abstracts in the testing corpus. CCNN and USE have fixed dimensions.

WE	$\mathbf{D}_{\mathrm{WE}}$	Model	#Nr	#Lr	mi$\mathbf{F_1}$	$\mathbf{T}_{\mathrm{t}}$
GloVe	4000 (50)	LSTM	128	2	0.71	620
		LSTM + Attn	128	2	0.72	689
		BiLSTM	128	2	0.73	676
		BiLSTM + Attn	128	2	**0.75**	801
		GRU	128	2	0.71	600
		GRU + Attn	128	2	0.73	591
		BiGRU	128	2	0.73	721
		BiGRU + Attn	128	2	0.74	740
		BiGRU	1280	2	0.74	1980
		BiGRU + Attn	1280	2	**0.75**	1300
		BiGRU	128	4	0.71	1012
		BiGRU + Attn	128	4	**0.75**	1144
SciBERT	61440 (768)	LSTM	128	2	0.70	619
		LSTM + Attn	128	2	0.69	431
		BiLSTM	128	2	0.72	949
		BiLSTM + Attn	128	2	0.71	557
		GRU	128	2	0.70	496
		GRU + Attn	128	2	0.69	412
		BiGRU	128	2	0.72	698
		BiGRU + Attn	128	2	0.71	537
FastText	8000 (100)	LSTM	128	2	0.65	560
		LSTM + Attn	128	2	**0.75**	748
		BiLSTM	128	2	0.68	658
		BiLSTM + Attn	128	2	**0.76**	862
		GRU	128	2	0.69	496
		GRU + Attn	128	2	0.74	752
		BiGRU	128	2	0.68	740
		BiGRU + Attn	128	2	**0.76**	852
CCNN	1014	CNN	1008	6	0.68	560
USE	512	MLP	1024	4	0.63	729
			2048	4	0.64	766
TFIDF + BoW	5000	SVM (RBF)	-	-	0.62	63537
TFIDF + BoW	5000	SVM (Linear)	-	-	0.61	3.1
TFIDF + BoW	5000	LR	-	-	0.66	3.8
TFIDF + BoW	5000	NB	-	-	0.20	5563
TFIDF + BoW	5000	RF	-	-	0.55	115
